# Gut Microbiota–Host Redox Crosstalk: Molecular Mechanisms, Disease Pathogenesis, and Therapeutic Interventions

**DOI:** 10.3390/antiox15101255

**Published:** 2026-10-01

**Authors:** Shuaihu Chen, Yulong Yin, Jie Yin, Xihong Zhou

**Affiliations:** 1 College of Veterinary Medicine, Hunan Agricultural University, Changsha 410128, China; 17867594001@163.com; 2 Institute of Subtropical Agriculture, The Chinese Academy of Sciences, Changsha 410125, China; yinyulong@isa.ac.cn; 3 Yuelushan Laboratory, Changsha 410128, China; 4 College of Animal Science and Technology, Hunan Agricultural University, Changsha 410128, China

**Keywords:** gut microbiota, oxidative stress, extracellular vesicles, intestine

## Abstract

The crosstalk between the gut microbiota and the host redox system is essential for maintaining intestinal homeostasis and systemic health. Disruption of this bidirectional interaction impairs redox balance, promotes oxidative stress, and contributes to the initiation and progression of a wide range of diseases, including inflammatory bowel disease, metabolic liver disease, neurodegenerative disorders, and chronic kidney disease. In this review, we comprehensively summarize the molecular mechanisms by which the gut microbiota regulates host redox homeostasis, focusing on microbial metabolites, immune signaling, and host antioxidant pathways. We also discuss emerging mechanisms underlying microbiota–host redox interactions, including extracellular vesicle (EV)-mediated interkingdom communication, microbiota-driven regulation of intestinal epithelial ferroptosis, and microbial metabolic reprogramming. Furthermore, we highlight how gut microbiota dysbiosis extends oxidative stress beyond the intestine through the gut–liver, gut–brain, and gut–kidney axes, thereby driving the development of multiple organ diseases. Finally, we summarize recent advances in microbiota-targeted therapies and engineered bacterial EVs as innovative strategies for restoring redox homeostasis, and discuss their potential for the precision prevention and treatment of oxidative stress-associated diseases as well as the development of next-generation microbiome-based therapeutics.

## 1. Introduction

Redox homeostasis is essential for cellular metabolism, tissue integrity, and organismal health. By maintaining a dynamic equilibrium between oxidant production and antioxidant defense, the redox system regulates key biological processes, such as cell proliferation, differentiation, and immune responses [1,2,3,4,5]. However, an overproduction of reactive oxygen species (ROS) or a compromised antioxidant capacity disrupts this balance, leading to oxidative stress (OS). Persistent OS promotes lipid peroxidation, protein oxidation, DNA damage, and mitochondrial dysfunction, ultimately contributing to cellular injury and disease development [6,7,8,9].

As the largest metabolic, digestive, and immune organ, the intestine is continuously exposed to dietary, microbial, and environmental stimuli, making it particularly susceptible to oxidative damage [10]. Intestinal epithelial cells are highly sensitive to redox imbalance, and sustained OS disrupts epithelial integrity, impairs barrier function, and triggers chronic inflammation, thereby driving the onset and progression of numerous intestinal disorders [11,12]. Increasing evidence also indicates that intestinal OS is not confined to the gut but can propagate to distant organs through systemic metabolic and immune networks, contributing to the pathogenesis of multiple chronic diseases. The gut microbiota is a central regulator of intestinal homeostasis and host physiology [13,14]. Beyond its established roles in nutrient metabolism and energy harvesting, the gut microbiota actively modulates host redox homeostasis through microbial metabolites, immune regulation, and intercellular communication, thereby enhancing antioxidant defenses, preserving epithelial barrier integrity, and limiting oxidative injury [12,15,16]. Conversely, the host shapes microbial composition and function by regulating the intestinal microenvironment, immune responses, and the release of extracellular vesicles (EVs), establishing a dynamic bidirectional crosstalk between the microbiota and the host [17,18,19]. Under pathological conditions, OS and gut microbiota dysbiosis reinforce each other in a self-perpetuating cycle, accelerating intestinal dysfunction and promoting systemic disease progression [20,21].

Recent advances have revealed that this microbiota–host redox crosstalk extends far beyond conventional metabolite-mediated signaling. Emerging mechanisms, including EV-mediated interkingdom communication, microbiota-dependent regulation of intestinal epithelial ferroptosis, and microbial metabolic reprogramming, have substantially expanded our understanding of how the gut microbiota influences host redox homeostasis and disease susceptibility. These discoveries also identify novel therapeutic targets for OS-related disorders. However, it should be emphasized that current evidence for these mechanisms is largely preclinical and, in many cases, associative; their causal roles in human disease and clinical efficacy remain to be established. In this review, we summarize the molecular mechanisms underlying gut microbiota–host redox crosstalk, with particular emphasis on microbial metabolites, EV-mediated communication, ferroptosis, and microbial metabolic reprogramming. We further discuss how OS is propagated through the gut–liver, gut–brain, and gut–kidney axes to drive multi-organ pathology, and highlight recent advances in microbiota-targeted interventions and engineered bacterial EVs (BEVs) as emerging therapeutic strategies. By integrating current evidence, this review provides a comprehensive framework for understanding microbiota-mediated redox regulation and offers perspectives for the development of targeted microbiome-based therapies for OS-related diseases. In addition, this review discusses the limitations of current evidence and outlines future research directions.

## 2. Theoretical Basis of Gut Microbiota–Host Redox System Interaction

### 2.1. Host Redox System and Oxidative Damage

The host redox system is a highly coordinated regulatory network that maintains the balance between oxidant generation and antioxidant defense. It encompasses ROS production and elimination, redox signal transduction, and the recognition and repair of oxidative damage, thereby preserving cellular homeostasis and normal physiological function [1,22]. A growing body of evidence indicates that this system also functions as a central interface through which the gut microbiota modulates host physiology.

#### 2.1.1. Oxidative Stress

ROS are continuously produced as byproducts of aerobic metabolism. Under physiological conditions, ROS serve as essential signaling molecules that regulate intracellular signal transduction [23], cell proliferation, and host immune defense [4,24]. However, excessive accumulation of ROS overwhelms antioxidant defenses, causing oxidative damage to lipids, proteins, and nucleic acids and ultimately resulting in OS [25]. OS is commonly assessed by measuring biomarkers of oxidative damage in tissues and biological fluids. These biomarkers primarily include lipid peroxidation products, such as malondialdehyde (MDA) and 4-hydroxynonenal (4-HNE) [26]; protein oxidation products, including protein carbonyls [27] and nitrotyrosine [28]; and DNA oxidation markers, represented by 8-hydroxy-2′-deoxyguanosine (8-OHdG) [29]. Collectively, these indicators provide an integrated evaluation of both local and systemic OS.

To maintain redox homeostasis, the host has evolved a sophisticated antioxidant defense system composed of enzymatic and non-enzymatic components [30,31]. The enzymatic antioxidant system primarily comprises superoxide dismutase (SOD), catalase (CAT), glutathione peroxidases (GPxs), and heme oxygenase-1 (HO-1) [32,33,34], which coordinately eliminate superoxide anions and hydrogen peroxide while maintaining protein thiol homeostasis. In contrast, the non-enzymatic antioxidant system centers on reduced glutathione (GSH), together with low-molecular-weight antioxidants such as L-ascorbic acid and vitamin E [35,36,37], which directly scavenge free radicals or act as essential cofactors for antioxidant enzymes [38,39].

#### 2.1.2. Key Redox Signaling Pathways

Redox homeostasis is orchestrated by several transcriptional signaling pathways, among which the nuclear factor erythroid 2-related factor 2 (Nrf2)–Kelch-like ECH-associated protein 1 (Keap1)–antioxidant response elements (ARE) and nuclear factor-κB (NF-κB) pathways are centrally. Under basal conditions, Nrf2 is sequestered in the cytoplasm through its interaction with Keap1, which facilitates Nrf2 ubiquitination and degradation, thereby maintaining low basal activity [40]. Oxidative or electrophilic stress induces oxidative modification of critical cysteine residues in Keap1, thereby disrupting the Nrf2–Keap1 complex and allowing Nrf2 [41,42,43] to translocate into the nucleus. Nuclear Nrf2 heterodimerizes with small Maf proteins and binds AREs in the promoters of target genes, thereby inducing the expression of antioxidant and cytoprotective genes, including HO-1, SOD, *glutamate–cysteine ligase catalytic subunit (GCLC)*, *glutamate–cysteine ligase modifier subunit (GCLM)*, and *NAD(P)H quinone dehydrogenase 1 (NQO1)* [42]. In parallel, NF-κB acts as a key transcriptional regulator linking OS to inflammation. Under resting conditions, NF-κB is retained in the cytoplasm by inhibitor of κBα (IκBα). OS activates IκB kinase (IKK), promoting IκBα phosphorylation and degradation, which releases NF-κB for nuclear translocation. Activated NF-κB subsequently induces the expression of pro-inflammatory cytokines, including TNF-α, IL-1β, and IL-6, thereby amplifying inflammatory responses and ROS production [43]. Importantly, extensive crosstalk exists between the Nrf2 and NF-κB pathways. Activation of Nrf2 suppresses NF-κB signaling by competing for the transcriptional coactivator p300/CBP and by inducing HO-1-derived anti-inflammatory mediators such as carbon monoxide. Conversely, sustained NF-κB activation can impair Nrf2-mediated antioxidant signaling, thereby exacerbating OS [44]. The dynamic balance between these two pathways is therefore critical for maintaining redox homeostasis.

Besides Nrf2 and NF-κB, other transcription factors also contribute to redox regulation. Forkhead box O3a (FOXO3a) functions as an important compensatory regulator under conditions of impaired Nrf2 signaling by promoting the expression of antioxidant enzymes such as manganese superoxide dismutase (MnSOD), CAT, and autophagy-related genes [45]. Moreover, hypoxia-inducible factor-1α (HIF1α) supports epithelial barrier integrity by promoting mucus production and epithelial repair in the physiologically hypoxic intestinal environment [46].

It is worth noting that microbiota–host redox crosstalk is not governed by a single pathway. TLR4–NF-κB/Nrf2 signaling is one important pathway, but its role varies with cell type, tissue microenvironment, and disease stage, and it interacts with pathways such as NADPH oxidases, the NOD-like receptor family pyrin domain containing 3 (NLRP3) inflammasome, ferroptosis, glutathione metabolism, and mitochondrial quality control.

Among these, mitochondria and the NADPH oxidase family (NOX1–5) serve as important sources of intracellular ROS, and their activities are regulated by microbial signals and inflammatory mediators [47,48]. Mitochondria-derived ROS and mitochondrial DNA release can further activate innate immune pathways [49,50], suggesting that mitochondria are both an important source of oxidative stress and a hub for its transmission to immune responses. Correspondingly, the host maintains redox balance through multi-layered scavenging and defense mechanisms. Glutathione metabolism is central to cellular antioxidant capacity, and the microbiota can regulate host GSH synthesis by affecting the metabolism of glutamine, glutamate, glycine, and cysteine [51,52]. When ROS scavenging is insufficient to counter ROS production, accumulated oxidative damage is converted into inflammatory signals: the NLRP3 inflammasome couples oxidative stress with IL-1β/IL-18 maturation, and excessive ROS can activate this pathway through the thioredoxin-interacting protein (TXNIP)/NLRP3 axis [53,54]. Meanwhile, mitophagy and mitochondrial quality control determine the clearance efficiency of damaged mitochondria, reducing sustained ROS accumulation at the source and maintaining epithelial barrier integrity [55,56]. These pathways intertwine with transcriptional networks such as Nrf2/NF-κB, collectively forming a complex redox regulatory network.

#### 2.1.3. Intestinal Epithelial Barrier Structure

The intestinal mucosal barrier is formed by a single layer of epithelial cells interconnected by tight junction complexes composed primarily of occludin, claudins, and zonula occludens (ZO) proteins. These structures enable efficient nutrient absorption while preventing the translocation of luminal pathogens and harmful molecules [57,58].

OS disrupts epithelial barrier integrity via multiple mechanisms. Excessive intracellular ROS activate matrix metalloproteinases (MMPs), leading to degradation of tight junction proteins, widening of intercellular spaces, and increased intestinal permeability, a pathological condition commonly referred to as “leaky gut” [59,60]. Once the barrier integrity is compromised, luminal components, including lipopolysaccharide (LPS), microbial toxins, and incompletely digested dietary macromolecules, can penetrate the epithelium and enter the lamina propria. These molecules then activate resident immune cells, particularly macrophages, triggering the production of pro-inflammatory cytokines such as TNF-α, IL-1β, and IL-6 [61]. These inflammatory mediators further stimulate nicotinamide adenine dinucleotide phosphate (NADPH) oxidases in intestinal epithelial cells, thereby increasing intracellular ROS generation and establishing a vicious cycle of OS and inflammation that contributes to the pathogenesis of inflammatory bowel disease (IBD) and other chronic intestinal disorders [62].

The oxidative damage, barrier disruption, and inflammatory amplification described above collectively create a hostile intestinal microenvironment in which the host antioxidant system alone is often insufficient to restore redox balance. Under these conditions, the gut microbiota becomes an essential contributor to intestinal redox homeostasis through its diverse metabolic activities and antioxidant mechanisms. Therefore, understanding the antioxidant functions of the gut microbiota is essential for elucidating the bidirectional interaction between the gut microbiota and the host redox system.

### 2.2. Antioxidant Characteristics of the Gut Microbiota

The intestine exhibits a steep oxygen gradient, which ranges from a relatively oxygen-rich environment in the lumen to a hypoxic or nearly anaerobic environment within the inner mucus layer adjacent to the epithelium [63]. This spatial heterogeneity shapes the ecological distribution of gut microorganisms and largely determines their distinct contributions to host redox regulation [64]. Based on differences in oxygen tolerance, metabolic characteristics, and endogenous antioxidant systems, gut microorganisms can be broadly classified into several functional groups with distinct redox-regulatory properties [20,65].

**Scheme 1:**
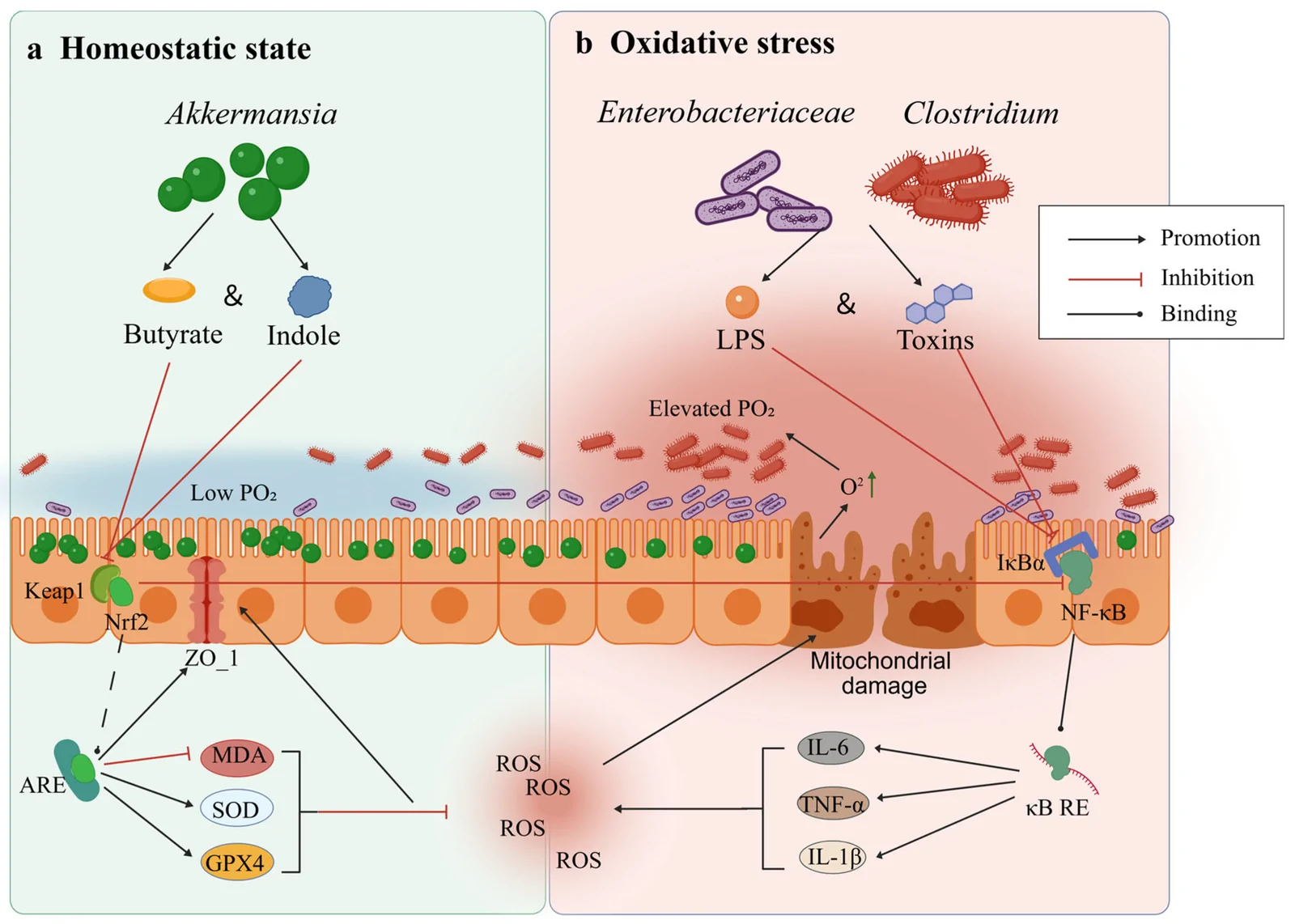
Gut microbiota maintains intestinal redox homeostasis. (**a**) Beneficial commensal bacteria produce metabolites, including butyrate and indole, that activate Nrf2–ARE signaling by inhibiting Keap1, enhance antioxidant defenses (SOD and GPX4), reduce lipid peroxidation, and maintain epithelial barrier integrity. (**b**) Gut microbiota dysbiosis promotes the expansion of pathogenic bacteria, which release LPS and toxins to activate NF-κB signaling, trigger inflammatory cytokine production and ROS generation, and induce oxidative epithelial injury. Increased epithelial oxygen leakage and luminal nitrate further favor the growth of facultative opportunistic pathogens, reinforcing a vicious cycle of dysbiosis, oxidative stress, and inflammation. ARE, antioxidant response element; GPX4, glutathione peroxidase 4; IL-1β, interleukin-1β; IL-6, interleukin-6; Keap1, Kelch-like ECH-associated protein 1; LPS, lipopolysaccharide; MDA, malondialdehyde; NF-κB, nuclear factor kappa B; Nrf2, nuclear factor erythroid 2-related factor 2; ROS, reactive oxygen species; SOD, superoxide dismutase; κB-RE, nuclear factor kappa B response element; ZO-1, zonula occludens-1.

Commensal obligate anaerobes, including members of the Lachnospiraceae family and *Akkermansia muciniphila*, predominantly colonize the hypoxic mucus layer and generally display limited tolerance to OS [66]. Rather than directly scavenging ROS, these microorganisms maintain intestinal redox homeostasis primarily through metabolite-mediated activation of host endogenous antioxidant-related pathways. For example, members of the Lachnospiraceae family produce short-chain fatty acids (SCFAs) such as butyrate through dietary fiber fermentation, while *Akkermansia muciniphila* produces propionate and acetate via mucin degradation, thereby cross-feeding butyrate-producing bacteria and indirectly promoting butyrate production; both groups also synthesize tryptophan-derived indole metabolites, which have been shown to activate host antioxidant signaling pathways, particularly the Nrf2 pathway, thereby enhancing epithelial antioxidant capacity and preserving intestinal homeostasis (Scheme 1a) [67,68].

In contrast, facultative anaerobes, represented by members of the Enterobacteriaceae, possess more robust endogenous antioxidant enzyme systems and therefore exhibit greater tolerance to oxygen and oxidative stress [69]. Under physiological conditions, these bacteria constitute only a minor proportion of the gut microbiota. However, OS disrupts epithelial oxygen consumption and increases luminal oxygen availability, thereby creating favorable conditions for their expansion. The overgrowth of facultative anaerobes promotes the release of LPS, which activates Toll-like receptor 4 (TLR4)-mediated NF-κB signaling in host cells, thereby amplifying inflammatory responses and ROS production and reinforcing a feed-forward cycle of oxidative stress and inflammation (Scheme 1b) [70,71].

Strict anaerobic opportunistic pathogens, exemplified by *Clostridium perfringens*, differ fundamentally from beneficial obligate anaerobes. Although *Clostridium perfringens* possesses a certain degree of oxidative tolerance [72], its pathogenicity is primarily attributed to the production of multiple exotoxins and virulence factors. Under homeostatic conditions, its abundance is tightly controlled by the resident commensal microbiota [73]. However, disruption of microbial homeostasis allows *Clostridium perfringens* to proliferate excessively and release toxins that impair mitochondrial structure and respiratory function in intestinal epithelial cells. The resulting mitochondrial dysfunction markedly increases mitochondrial ROS production, thereby aggravating epithelial oxidative injury and intestinal inflammation (Scheme 1b) [74,75].

Importantly, the antioxidant functions of the gut microbiota are highly strain-dependent. Even within the same bacterial species, substantial differences in antioxidant capacity and redox-regulatory activity have been reported. Genetic variation, adaptive evolution, host physiological status, oxygen availability, and nutrient composition can all influence microbial metabolism and functional outputs, contributing to considerable inter-strain heterogeneity in host redox regulation [76,77].

### 2.3. Gut Microbiota–Host Redox Interaction

The gut microbiota and the host redox system engage in a dynamic and reciprocal interaction that is essential for maintaining intestinal homeostasis. On the one hand, alterations in the host redox environment reshape microbial community structure and metabolic function. On the other hand, the gut microbiota modulates host redox homeostasis through the production of bioactive metabolites and BEVs, thereby influencing OS, inflammation, and epithelial barrier integrity.

#### 2.3.1. Remodeling of the Gut Microbiota by Host OS

Host OS profoundly alters the intestinal microenvironment, thereby reshaping the composition and function of the gut microbiota [78]. During intestinal inflammation, mitochondrial oxidative phosphorylation in intestinal epithelial cells is impaired, which reduces epithelial oxygen consumption and increasing oxygen diffusion into the intestinal lumen [79]. Simultaneously, inflammatory responses promote the generation of alternative electron acceptors, particularly nitrate, creating favorable conditions for the expansion of facultative anaerobic bacteria [80]. The combined increase in luminal oxygen availability and nitrate concentration selectively favors facultative anaerobes while suppressing obligate anaerobic commensals that thrive in low-oxygen environments, ultimately leading to microbial dysbiosis [81].

Microbial dysbiosis is accompanied by profound metabolic reprogramming [82]. The abundance of pathways responsible for the production of beneficial metabolites, including butyrate and indole derivatives, is markedly reduced, whereas pathways associated with LPS biosynthesis, branched-chain amino acid metabolism, and other pro-inflammatory processes become enriched [83,84,85,86]. Consequently, the microbiota exhibits diminished antioxidant potential and an increased capacity to promote inflammation, thereby reinforcing epithelial OS and establishing a self-perpetuating cycle of oxidative damage and intestinal inflammation.

Persistent OS may also induce long-term functional adaptation of the gut microbiota [20]. Continuous exposure to an oxidative environment can alter microbial gene expression and metabolic activity, enabling some obligate anaerobes to survive despite unfavorable conditions. However, these adaptive responses are frequently accompanied by reduced production of beneficial metabolites, particularly butyrate. As a result, restoration of microbial metabolic function often lags behind the resolution of inflammation, which may contribute to the persistence or recurrence of chronic intestinal disorders [87,88].

#### 2.3.2. Regulation of Host Redox Homeostasis by the Gut Microbiota

Conversely, the gut microbiota actively contributes to host redox homeostasis through the production of bioactive metabolites and BEVs. These microbial signals regulate OS primarily through three complementary mechanisms: modulation of redox signaling pathways, maintenance of intestinal barrier integrity, and regulation of mucosal immune responses.

Among microbial metabolites, short-chain fatty acids, tryptophan-derived metabolites, and secondary bile acids are the most extensively characterized antioxidants. Short-chain fatty acids such as butyrate alleviate oxidative stress by reducing ROS and MDA levels and enhancing endogenous antioxidant enzyme activity. This effect has been demonstrated in intestinal epithelial cell models. In rotavirus-infected IPEC-J2 cells, sodium butyrate activates the G protein-coupled receptor 109A (GPR109A) receptor-mediated AMPK-Nrf2 signaling pathway, increases SOD, CAT, and GPxsactivities, and upregulates HO-1 and NQO1 protein levels [89]. In addition, butyrate supports mitochondrial oxidative phosphorylation, thereby maintaining colonocyte energy metabolism and limiting mitochondrial ROS generation [90]. Tryptophan-derived metabolites also alleviate oxidative stress and improve intestinal barrier function; in a pseudo-germ-free mouse colitis model, metabolites such as indole-3-propionic acid and indole-3-acetic acid produced by *Lactiplantibacillus plantarum* Y42 from tryptophan enhance colonic antioxidant-related defense and improve barrier function by activating endogenous antioxidant pathways (aryl hydrocarbon receptor, AhR and Nrf2 signaling) pathways and attenuating inflammation (NF-κB inhibition) [91]. Secondary bile acids indirectly reduce oxidative stress by activating Takeda G protein-coupled receptor 5 (TGR5) and farnesoid X receptor (FXR), suppressing NF-κB- and NLRP3-mediated inflammatory signaling [92].

The gut microbiota also preserves intestinal redox balance by maintaining epithelial barrier integrity. Microbial metabolites, particularly butyrate, serve as the primary energy source for colonocytes, promoting epithelial renewal and enhancing the expression of tight junction proteins, thereby strengthening epithelial barrier function [93]. Furthermore, in T84 and Caco-2 polarized monolayer cell models, BEVs and soluble factors released by the probiotic *Escherichia coli* Nissle 1917 and the commensal *Escherichia coli* ECOR63 upregulate ZO-1 and claudin-14 while downregulating claudin-2, thereby enhancing epithelial barrier function [94]. These barrier-protective effects help limit the translocation of luminal antigens and toxins, thereby reducing oxidative stress and inflammation. In contrast, pathogen-derived BEVs can disrupt tight junctions; for example, BEVs from *Vibrio cholerae* A1552 degrade claudin, ZO-1, and β-catenin through the zinc-dependent metalloprotease HapA [95], leading to intestinal disruption and exacerbated oxidative stress.

In addition, the gut microbiota regulates redox homeostasis through modulation of mucosal immunity [96]. In macrophage models, butyrate secreted by the microbiota suppresses excessive M1 macrophage activation while promoting anti-inflammatory M2 polarization, thereby increasing IL-10 production and reducing the secretion of pro-inflammatory and pro-oxidative mediators such as IL-1β and inducible nitric oxide synthase (iNOS) [97]. Butyrate also inhibits dendritic cell maturation and promotes type 1 regulatory T (Tr1) cell differentiation through dendritic cell regulation [98]; furthermore, in mouse models, butyrate promotes extrathymic regulatory T cell (Treg) differentiation [99]. These immunomodulatory effects collectively establish an anti-inflammatory immune environment and alleviate intestinal oxidative stress by reducing the production of pro-oxidative mediators such as iNOS and pro-inflammatory cytokines.

Collectively, microbial metabolites, epithelial barrier maintenance, and immune regulation function in a coordinated manner to preserve host redox homeostasis. Disruption of any of these interconnected processes may contribute to sustained OS and the development of intestinal diseases (Scheme 1).

## 3. Recent Advances in Gut Microbiota–Host Redox Interaction

Recent years have witnessed rapid advances in understanding the bidirectional interaction between the gut microbiota and the host redox system. Emerging evidence indicates that BEV-mediated interkingdom communication, microbiota-regulated ferroptosis of intestinal epithelial cells, and microbial metabolic reprogramming represent three major frontiers in this field.

### 3.1. EV-Mediated Microbiota–Host Communication

EVs are nanosized membrane-enclosed particles secreted by both microorganisms and host cells that mediate intercellular communication through the delivery of bioactive cargoes. According to their cellular origin, EVs can be broadly classified into BEVs and host-derived EVs, of which exosomes (Exos) are a specific biogenesis subtype [100]. BEVs comprise vesicles secreted by both Gram-negative and Gram-positive bacteria. In Gram-negative bacteria, these vesicles are typically referred to as outer membrane vesicles (OMVs) because they originate from the outer membrane. BEVs contain diverse bioactive molecules, including LPS, membrane proteins, lipids, nucleic acids, enzymes, and small metabolites, enabling direct communication between the microbiota and host tissues [101]. In contrast, Exos are generated through fusion of multivesicular bodies with the plasma membrane and transport a wide range of functional molecules, including microRNAs, proteins, lipids, and metabolites, thereby mediating communication between host cells [102].

#### 3.1.1. Regulation of Host Intestinal OS by BEVs

BEVs released by gut microorganisms can penetrate the intestinal mucus layer, enter epithelial cells, and modulate host redox homeostasis. Their biological effects largely depend on their microbial origin: BEVs derived from commensal bacteria generally exhibit antioxidant-related and barrier-protective properties, whereas those released by pathogenic bacteria frequently promote OS and intestinal injury [103].

Commensal BEVs encapsulate a variety of bioactive cargos, including proteins, polysaccharides, nucleic acids, and small peptides [104]. These vesicles are internalized by intestinal epithelial cells through endocytosis or membrane fusion, where they regulate intracellular signaling pathways [105]. For example, in an LPS-induced Caco-2 intestinal epithelial cell model in vitro, BEVs derived from *Akkermansia muciniphila* (AmEVs) downregulated TIR-domain-containing adapter-inducing interferon-β (TRIF), myeloid differentiation primary response 88 (MyD88), p38 mitogen-activated protein kinase (MAPK), and Fos proto-oncogene, AP-1 transcription factor subunit (FOS) protein levels while upregulating transforming growth factor beta receptor 2 (TGFBR2), thereby inhibiting MAPK signaling, reducing ROS and MDA levels, restoring catalase activity, and suppressing the expression of pro-inflammatory cytokines TNF-α, IL-1β, and IL-6. Furthermore [106], in a high-fat diet (HFD)-induced diabetic mouse model, AmEVs upregulated the expression of tight junction proteins such as occludin by activating AMPK signaling, thereby enhancing epithelial barrier integrity [107]. In contrast, BEVs derived from pathogenic bacteria frequently contain LPS, hemolysins, virulence factors, and other pro-inflammatory molecules [108]. Following cellular uptake, these cargos activate TLR4-dependent inflammatory signaling, leading to NF-κB activation, excessive ROS production, and oxidative injury. Furthermore, pathogen-derived BEVs can impair mitochondrial membrane potential and mitochondrial respiration, thereby increasing electron leakage and mitochondrial ROS generation [109,110]. These events contribute to sustained epithelial oxidative damage and chronic intestinal inflammation.

#### 3.1.2. Regulation of the Gut Microbiota by Host-Derived Exos

Communication between the microbiota and the host is bidirectional. In addition to microbiota-derived BEVs regulating host physiology, host-derived EVs have been proposed to participate in microbiota–host communication. Under conditions of OS or intestinal inflammation, intestinal epithelial cells can release Exos carrying functional miRNAs and other cargoes into the intestinal lumen, and these Exos have been implicated in the regulation of mucosal inflammation [111]. Host-derived EVs and their miRNA cargoes have also been implicated in microbiota–host crosstalk in intestinal diseases [112]. In addition, microbiota-derived LPS can stimulate NF-κB signaling and induce host miR-146a and miR-155, suggesting a potential route by which microbial signals modulate host miRNA responses [113]. However, the above evidence mainly supports a role for endogenous intestinal epithelial Exos in mucosal immunity and inflammation, and does not establish that they directly regulate gut microbial composition or function. In contrast, exogenous dietary/milk-derived Exos have been more extensively studied for their ability to regulate the gut microbiota. For example, incubation of human breast milk-derived Exos with infant gut microbiota significantly reduced the abundance of opportunistic pathogens, including *Escherichia coli*, *Klebsiella pneumoniae*, and *Shigella flexneri*, while increasing beneficial bacteria such as *Bacteroides fragilis* and *Bifidobacterium pseudocatenulatum*; these microbial changes were accompanied by increased production of SCFAs and indole metabolites, suggesting improved microbial metabolic function [114]. Animal studies also showed that oral administration of bovine milk-derived Exos restored gut microbial diversity in dextran sulfate sodium (DSS)-induced colitis mice, increased the abundance of beneficial taxa such as *Roseburia*, and promoted recovery from intestinal inflammation [115]. These studies indicate that exogenous dietary/milk-derived Exos can modulate gut microbial composition and metabolic activity, although the evidence is mainly derived from in vitro and animal models and requires further validation.

Overall, current evidence suggests that exogenous dietary/milk-derived Exos have the potential to modulate the gut microbiota, whereas direct evidence that endogenous intestinal epithelial cell-derived Exos regulate the gut microbiota in vivo remains lacking. Future studies using approaches such as intestinal epithelial cell-specific exosome labeling and analyses of intestinal epithelial exosome–microbiota interactions are needed to define the causal role of endogenous intestinal epithelial Exos in gut microbiota regulation and host redox homeostasis.

### 3.2. Microbiota-Regulated Intestinal Epithelial Ferroptosis

Ferroptosis is a regulated form of cell death characterized by iron-dependent lipid peroxidation. It occurs when the production of phospholipid hydroperoxides exceeds the capacity of cellular antioxidant systems, resulting in catastrophic membrane damage and cell death [116]. Increasing evidence suggests that the gut microbiota influences intestinal epithelial ferroptosis primarily by regulating iron homeostasis and antioxidant defense mechanisms.

#### 3.2.1. Regulation of Iron Homeostasis by the Gut Microbiota

Iron is an essential micronutrient for both the host and intestinal microorganisms. While a proportion of dietary iron is absorbed in the duodenum and proximal jejunum, unabsorbed iron reaches the colon, where it serves as an important nutrient source for the gut microbiota [117]. Under physiological conditions, certain commensal bacteria, particularly *Lactobacillus* spp., produce metabolites such as 1,3-diaminopropane (DAP) and reuterin, which have been reported to modulate epithelial iron transport by suppressing the expression of divalent metal transporter 1 (DMT1), thereby limiting intestinal iron uptake and contributing to systemic iron homeostasis [118]. In germ-free mouse models, loss of the gut microbiota is accompanied by upregulated duodenal DMT1 expression, reduced iron absorption, decreased expression of the iron storage protein ferritin and the basolateral iron exporter ferroportin, leading to increased iron retention in intestinal epithelial cells [118,119].Excess intracellular ferrous iron catalyzes the Fenton reaction, generating highly reactive hydroxyl radicals that initiate lipid peroxidation and increase susceptibility to ferroptosis.

In addition to regulating host iron transport, some gut bacteria, particularly members of the Enterobacteriaceae, actively compete with the host for iron through the secretion of siderophores. These high-affinity iron-chelating molecules capture luminal iron and transport it into bacterial cells via specific uptake systems [117,120]. Although this strategy promotes bacterial survival, it may also alter local iron availability and influence microbial community composition by limiting iron access for competing commensal microorganisms [118].

#### 3.2.2. Regulation of Antioxidant Defense by the Gut Microbiota

**Scheme 2:**
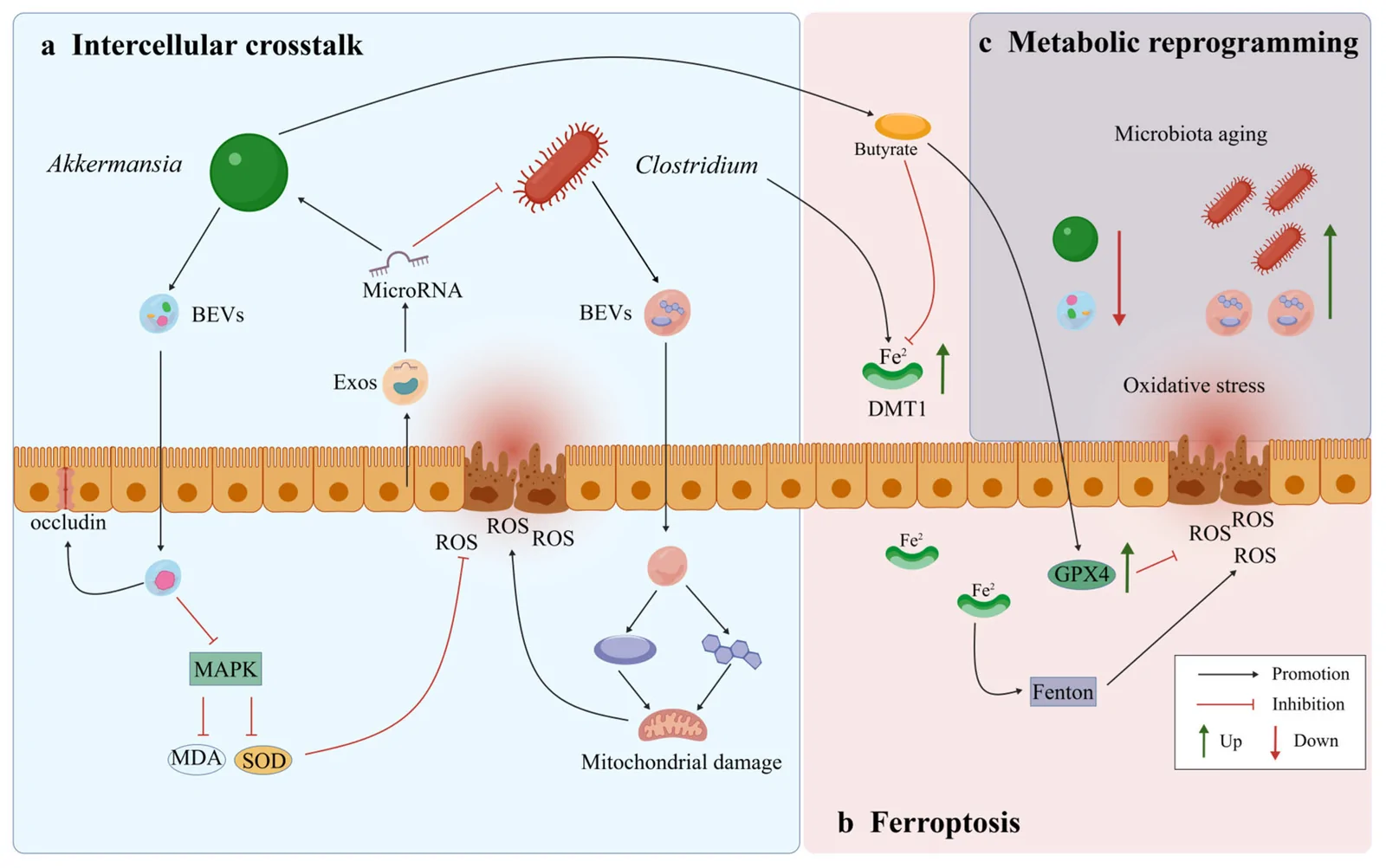
Mechanisms underlying microbiota–host redox crosstalk. (**a**) BEVs and host-derived EVs (e.g., Exos) mediate bidirectional interkingdom communication. Beneficial commensal-derived BEVs reinforce epithelial barrier integrity and antioxidant defenses, whereas pathogenic BEVs induce mitochondrial dysfunction and ROS accumulation. Host-derived EVs reciprocally regulate gut microbiota composition and function. (**b**) Opportunistic pathogens promote iron-dependent ROS production and lipid peroxidation, triggering intestinal epithelial ferroptosis. By contrast, butyrate-producing bacteria enhance GPX4 expression through butyrate production, thereby suppressing lipid peroxidation and ferroptotic cell death. (**c**) Oxidative stress and aging induce microbiota metabolic reprogramming, characterized by the loss of beneficial commensals and expansion of opportunistic pathogens, which further amplifies redox imbalance and intestinal dysfunction. DMT1, divalent metal transporter 1; Exos, exosomes; Fe^2+^, ferrous iron; GPX4, glutathione peroxidase 4; MDA, malondialdehyde; BEVs, bacterial extracellular vesicles; ROS, reactive oxygen species; SOD, superoxide dismutase.

Beyond iron metabolism, the gut microbiota modulates ferroptosis by regulating host antioxidant systems that detoxify lipid peroxides. Among these, the GSH-GPX4 axis represents the best-characterized protective pathway. GPX4 is a selenium-dependent phospholipid hydroperoxidase that reduces lipid hydroperoxides to non-toxic lipid alcohols using GSH as an essential electron donor. Tryptophan-derived metabolites such as indole-3-propionic acid activate the AhR, thereby supporting the synthesis of GSH and reinforcing intracellular antioxidant capacity [121,122]. In a DSS-induced colitis mouse model, tryptophan metabolism also inhibits intestinal epithelial ferroptosis by activating AhR/Nrf2 signaling and upregulating GPX4 expression. Conversely, microbial dysbiosis is frequently associated with depletion of tryptophan-metabolizing bacteria, resulting in impaired Nrf2 and AhR signaling, reduced GPX4 expression, insufficient GSH synthesis, and excessive accumulation of lipid peroxides. These alterations increase the susceptibility of intestinal epithelial cells to ferroptosis and compromise intestinal barrier integrity (Scheme 2b) [123].

In addition to the canonical GPX4 pathway, several GPX4-independent ferroptosis defense systems have recently been identified, including the ferroptosis suppressor protein 1 (FSP1)-coenzyme Q10 (CoQ10) pathway and the dihydroorotate dehydrogenase (DHODH)-CoQ10 pathway [124,125]. FSP1, localized to the plasma membrane, and DHODH, localized to the inner mitochondrial membrane, both catalyze the reduction of CoQ10 to ubiquinol, which functions as a potent lipid radical scavenger to suppress ferroptosis at distinct subcellular locations [126,127]. However, most current studies on the regulation of ferroptosis by gut microbial metabolites have focused on the aforementioned GPX4-dependent ferroptosis defense pathways. The molecular mechanisms linking microbial metabolites to these GPX4-independent ferroptosis defense pathways remain unclear and warrant further investigation.

### 3.3. Microbiota Metabolic Reprogramming and Redox Regulation

Microbial metabolites are central mediators of the interaction between the gut microbiota and the host redox system. Importantly, the metabolic activity of the gut microbiota is highly dynamic rather than static. In response to environmental perturbations such as oxidative stress and aging, the microbiota undergoes metabolic reprogramming, leading to altered production of bioactive metabolites that ultimately influence host redox homeostasis.

#### 3.3.1. Metabolic Reprogramming Under OS

OS induces rapid alterations in both the composition and metabolic function of the gut microbiota. Although transient metabolic adaptation may initially help maintain microbial survival, persistent OS ultimately disrupts microbial homeostasis and reshapes microbial metabolic outputs. In DSS-induced acute and chronic colitis mouse models, oxidative stress is accompanied by marked alterations in gut microbiota composition, manifested as a significant decrease in the abundance of butyrate-producing bacteria such as members of the Lachnospiraceae and Ruminococcaceae families. In vitro culture experiments further validated this pattern: oxidative stress reduced the abundance of butyrate-producing bacteria (such as *Agathobacter* and *Anaerostipes*) and total butyrate levels, whereas facultative anaerobes such as *Escherichia-Shigella* and *Enterococcus* exhibited greater tolerance [20]. These changes may reduce the production of microbial metabolites with antioxidant potential, such as butyrate, thereby weakening the activation of host antioxidant defense pathways and potentially shifting the functional phenotype of the gut microbiota from a homeostatic maintenance type toward a pro-oxidative, pro-inflammatory type (Scheme 2c).

#### 3.3.2. Age-Associated Metabolic Reprogramming of the Gut Microbiota

Aging is accompanied by progressive remodeling of the gut microbiota, a process commonly referred to as age-associated dysbiosis. This process is generally characterized by reduced microbial diversity [128] together with diminished abundance of beneficial microorganisms involved in the production of butyrate and tryptophan-derived metabolites [129]. As a consequence, the metabolic capacity of the aging microbiota gradually shifts toward a less antioxidant-supportive profile. Longitudinal studies have demonstrated that aging is associated with alterations in the abundance of several dominant bacterial taxa. For example, the abundance of Lachnospiraceae generally declines with age, whereas specific members of the Ruminococcaceae exhibit variable age-dependent changes [130]. Correspondingly, intestinal concentrations of butyrate and indole metabolites are significantly lower in older individuals than in younger adults [129]. In parallel, expression of microbial genes encoding butyryl-CoA:acetate CoA-transferase, the key enzyme responsible for butyrate synthesis, also decreases during aging [131]. Age-associated depletion of butyrate-producing bacteria is frequently accompanied by an increased abundance of facultative anaerobic bacteria, including members of the Enterobacteriaceae, resulting in elevated production of LPS and other pro-inflammatory metabolites [132]. These metabolic alterations may contribute to chronic low-grade inflammation and sustained oxidative stress, thereby accelerating the depletion of endogenous antioxidant defenses and disrupting systemic redox homeostasis (Scheme 2c).

## 4. Mechanisms of OS-Mediated Intestinal and Systemic Organ Injury

The reciprocal amplification of gut microbiota dysbiosis and OS not only drives local intestinal injury but also facilitates the dissemination of oxidative and inflammatory signals to distant organs. Through microbial metabolites, BEVs, LPS, and circulating inflammatory mediators, intestinal OS propagates along the gut–liver, gut–brain, and gut–kidney axes, contributing to systemic oxidative damage and the pathogenesis of multiple chronic diseases.

### 4.1. Local Intestinal Oxidative Injury

#### 4.1.1. Inflammatory Bowel Disease

IBD, including ulcerative colitis (UC) and Crohn’s disease (CD), is characterized by chronic relapsing intestinal inflammation that arises from the interaction of genetic susceptibility, immune dysregulation, environmental factors, and gut microbiota dysbiosis. A hallmark of IBD is a self-reinforcing cycle that links microbial dysbiosis, oxidative stress, epithelial injury, and chronic inflammation [133]. During disease progression, excessive ROS disrupt epithelial tight junctions by reducing the expression of proteins such as occludin and ZO-1, thereby increasing intestinal permeability [134]. OS also promotes ferroptosis of intestinal epithelial cells, further compromising barrier integrity [135]. The resulting translocation of bacteria and microbial products into the lamina propria activates innate immune responses, leading to sustained production of pro-inflammatory cytokines that further amplify OS and perpetuate chronic intestinal inflammation [133].

#### 4.1.2. Colorectal Cancer

Persistent oxidative stress is also recognized as an important contributor to colorectal cancer (CRC) development. Chronic ROS accumulation induces oxidative DNA damage, particularly the formation of 8-hydroxy-2′-deoxyguanosine (8-OHdG), thereby increasing mutation rates in tumor suppressor genes such as *APC* and *TP53* and promoting malignant transformation [136]. Compared with healthy individuals, patients with CRC typically exhibit depletion of butyrate-producing bacteria, including members of the Lachnospiraceae and Ruminococcaceae, together with enrichment of Enterobacteriaceae and *Fusobacterium nucleatum*. These microbial alterations are accompanied by elevated intestinal OS, as reflected by increased MDA and 8-OHdG levels [137,138]. Moreover, reduced butyrate availability alters epithelial energy metabolism and may impair normal regulation of cell proliferation and differentiation [139]. Although oxidative stress contributes to both IBD and CRC, its pathological role differs between the two diseases. In IBD, oxidative injury primarily serves as a consequence and amplifier of inflammation, whereas in CRC, persistent oxidative DNA damage and microbiota-associated metabolic alterations are considered major drivers of tumor initiation and progression [140].

### 4.2. Gut–Organ Axis-Mediated Transmission of Oxidative Injury

**Scheme 3:**
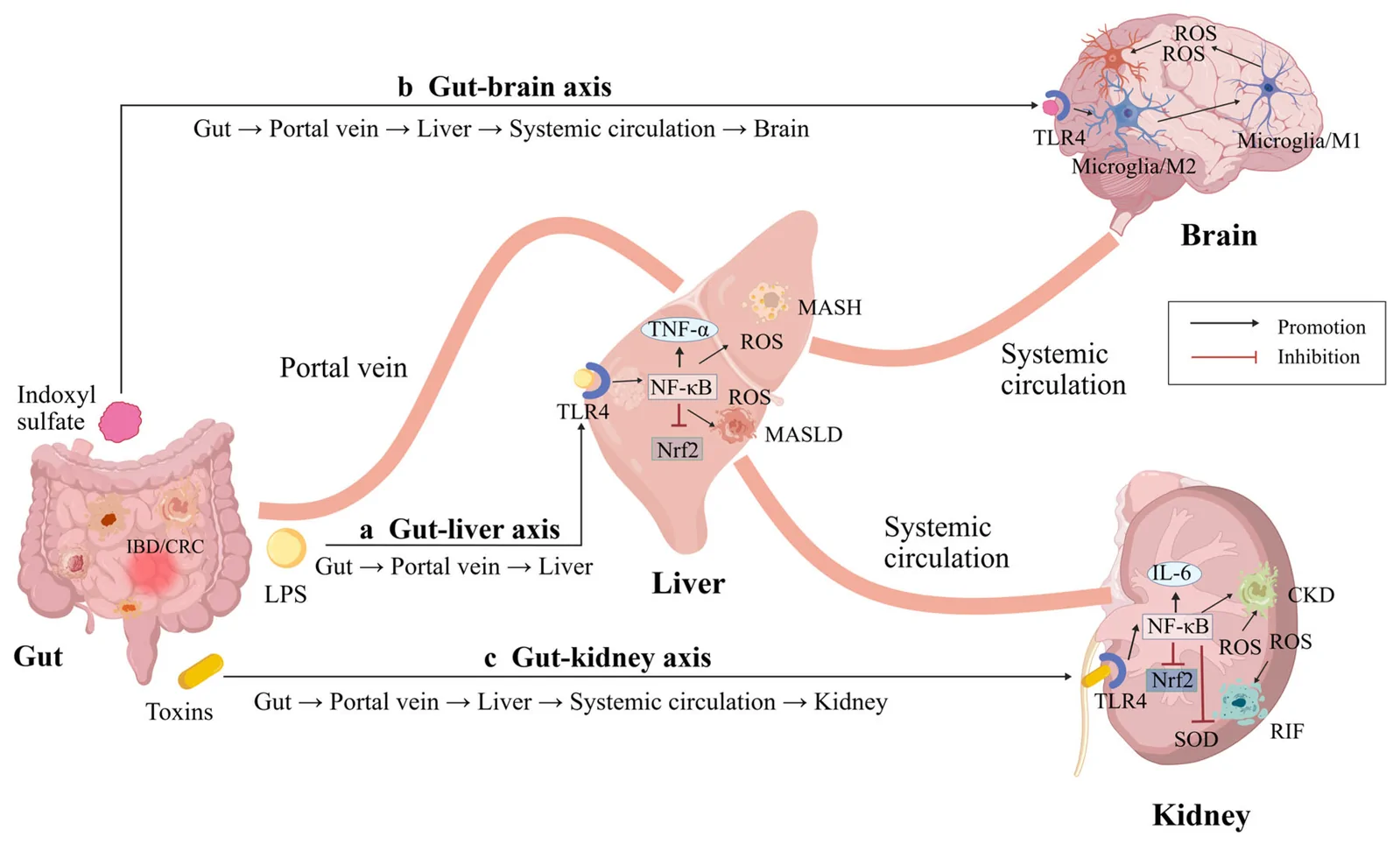
Systemic propagation of oxidative stress through gut–organ axes. (**a**) Gut–liver axis. Gut-derived LPS and microbial metabolites activate hepatic TLR4–NF-κB signaling, suppress Nrf2-dependent antioxidant responses, and promote oxidative stress, contributing to the progression of MASLD and MASH. (**b**) Gut–brain axis. Microbial metabolites enter the circulation, cross the blood–brain barrier, activate microglial TLR4–NF-κB signaling, and induce neuroinflammation and oxidative neuronal damage. (**c**) Gut–kidney axis. Gut microbial metabolites are converted into IS in the liver. Circulating IS activates renal TLR4–NF-κB signaling, impairs antioxidant defenses, and aggravates oxidative kidney injury. CKD, chronic kidney disease; CRC, colorectal cancer; IBD, inflammatory bowel disease; IL-6, interleukin-6; M1, M1-type microglia/macrophage; M2, M2-type microglia/macrophage; MASLD, metabolic dysfunction-associated steatotic liver disease; MASH, metabolic dysfunction-associated steatohepatitis; NF-κB, nuclear factor kappa B; Nrf2, nuclear factor erythroid 2-related factor 2; ROS, reactive oxygen species; RIF, renal interstitial fibrosis; SOD, superoxide dismutase; TLR4, Toll-like receptor 4; TNF-α, tumor necrosis factor-α; κB-RE, nuclear factor kappa B response element.

Disruption of intestinal redox homeostasis is not restricted to the gut. Increased intestinal permeability permits microbial metabolites, LPS, BEVs, and inflammatory mediators to enter the circulation through the portal vein, lymphatic system, and systemic blood flow. These circulating signals subsequently influence distant organs via the gut–liver, gut–brain, and gut–kidney axes, thereby contributing to systemic OS and chronic disease progression [141,142]. Despite the distinct characteristics of individual gut–organ axes, they share several common pathogenic mechanisms underlying oxidative injury transmission (Scheme 3). These mechanisms include disruption of the intestinal epithelial barrier, increased systemic exposure to microbial-derived molecules, activation of inflammatory signaling pathways, mitochondrial dysfunction, and impairment of endogenous antioxidant defenses. Collectively, these processes establish a continuous communication network between the intestine and peripheral organs, through which intestinal OS can be amplified and propagated throughout the body.

#### 4.2.1. Gut–Liver Axis

Among gut–organ communication pathways, the gut–liver axis is the most extensively characterized because the liver receives portal venous blood directly from the intestine, making it the first organ exposed to gut-derived microbial products. During intestinal OS, increased intestinal permeability facilitates the translocation of LPS, oxidized lipids, and other microbial products into the portal circulation. In the liver, LPS activates TLR4 signaling in hepatocytes and resident macrophages, promoting NF-κB-mediated inflammatory responses while suppressing Nrf2-dependent antioxidant defenses [48]. The pathogenic role of this inflammation–antioxidant imbalance pathway has been validated by in vivo intervention studies: in an LPS-induced liver injury mouse model, sodium humate (HNa) pretreatment inhibited TLR4/NF-κB signaling, enhanced hepatic M2 macrophage polarization, and reduced inflammatory cytokine levels, thereby ameliorating liver pathological injury and inflammation; in parallel, HNa enhanced hepatic antioxidant capacity and reduced hepatocyte apoptosis by activating Nrf2/HO-1 signaling [143]. The resulting imbalance accelerates ROS accumulation, lipid peroxidation, and mitochondrial dysfunction (Scheme 3a). Clinical observations also support the association between intestinal barrier damage and liver pathology. A prospective cross-sectional study including 72 metabolic dysfunction-associated steatotic liver disease (MASLD) patients and 68 healthy controls showed that serum LPS and intestinal fatty acid-binding protein (I-FABP) levels were significantly elevated in MASLD patients, and LPS was positively correlated with liver stiffness and controlled attenuation parameter (CAP) scores, while I-FABP was positively correlated with liver stiffness; LPS was also positively correlated with C-reactive protein (CRP) levels. The study also found no significant difference in occludin between groups, suggesting that it may not be a reliable marker for assessing intestinal barrier function in MASLD [144]. Furthermore, in a metabolic dysfunction-associated steatohepatitis (MASH) mouse model, soluble sialic acid-binding immunoglobulin-like lectin 9 (sSiglec-9) treatment reduced intestinal permeability and liver inflammation, and significantly improved the MASLD activity score, which was further confirmed in in vitro cell experiments [145]. Persistent delivery of gut-derived oxidative signals contributes to hepatocyte lipid accumulation, activation of hepatic stellate cells, extracellular matrix deposition, and chronic hepatic inflammation, thereby promoting the progression of metabolic dysfunction-associated steatotic liver disease (MASLD; formerly NAFLD), metabolic dysfunction-associated steatohepatitis (MASH; formerly NASH), liver fibrosis, and ultimately cirrhosis [146].

#### 4.2.2. Gut–Brain Axis

The gut–brain axis integrates neural, endocrine, immune, and metabolic signaling pathways, with the gut microbiota serving as a central regulator. Increasing evidence indicates that intestinal OS influences brain function through circulating microbial metabolites, inflammatory mediators, and bacterial extracellular vesicles [147]. Certain gut microbiota-derived metabolites, such as trimethylamine N-oxide (TMAO), p-cresyl sulfate (pCS), 4-ethylphenyl sulfate (4-EPS), and indoxyl sulfate (IS), can influence blood–brain barrier integrity or enter the central nervous system under pathological conditions, where they promote microglial activation through pathways including TLR4 and the aryl hydrocarbon receptor (AhR) [147,148]. Animal model intervention studies provide clues for this pathway: in heart failure mice, increased intestinal permeability and elevated plasma LPS levels were accompanied by increased brain TLR4 expression and exacerbated neuroinflammation; administration of the intestinal barrier protectant intestinal alkaline phosphatase (IAP) or TLR4 knockout significantly alleviated neuroinflammation [149]. Activated microglia generate excessive ROS and pro-inflammatory cytokines, leading to neuronal oxidative injury, synaptic dysfunction, and neuroinflammation (Scheme 3b) [148,149]. If such gut OS-driven neuroinflammation persists long-term, it may further contribute to chronic neurodegenerative pathology. These findings suggest that the gut–brain axis is a potential intervention target for neurological diseases, although its specific mechanisms and clinical translational value require further investigation.

#### 4.2.3. Gut–Kidney Axis

Communication along the gut–kidney axis is largely mediated by gut-derived uremic toxins, inflammatory mediators, and oxidative metabolites [150]. During microbial dysbiosis, bacterial metabolism produces increased amounts of protein-derived uremic toxins, including IS and pCS. Following hepatic metabolism, these compounds accumulate in the circulation and are transported to the kidneys [150,151]. Within renal tubular epithelial cells, these toxins activate NF-κB signaling while suppressing Nrf2-mediated antioxidant responses, leading to excessive ROS production and oxidative injury. Persistent oxidative stress promotes tubular epithelial damage, fibroblast activation, extracellular matrix deposition, and renal interstitial fibrosis, thereby accelerating the progression of chronic kidney disease (CKD) (Scheme 3c) [152]. Importantly, CKD further aggravates gut dysbiosis by reducing the renal clearance of circulating uremic toxins. The resulting toxin accumulation further disrupts intestinal microbial homeostasis and enhances oxidative stress, establishing a feed-forward cycle linking microbial dysbiosis, oxidative injury, toxin retention, and progressive renal dysfunction [151,152,153].

To facilitate comparison of the strength of evidence and causal inference for the mechanisms discussed in Section 3 and Section 4, Table 1 summarizes their predominant evidence sources, representative models/populations, key limitations, and representative references.

As shown in Table 1, the current evidence for the mechanisms discussed in Section 3 and Section 4 is mainly derived from in vitro and animal studies, with limited human causal evidence; some pathways remain associative, and there are clear limitations in causal inference, model translation, and clinical validation. Future studies should further strengthen human interventional research and causal validation.

## 5. Targeted Regulation of Gut Microbiota–Host OS Interaction

Targeted modulation of the gut microbiota represents a potential strategy for restoring host redox homeostasis. Current approaches can be broadly classified into two major categories: microbiota remodeling strategies, which aim to reconstruct a beneficial microbial ecosystem by altering community composition and metabolic capacity, and microbial signal-based strategies, which enhance microbiota–host communication through functional molecules such as extracellular vesicles. These approaches provide complementary opportunities for alleviating OS-associated intestinal and systemic disorders.

### 5.1. Targeted Remodeling of Gut Microbiota Structure

The fundamental principle of microbiota remodeling is to selectively reshape microbial community structure by enriching beneficial microorganisms with antioxidant functions, such as butyrate- and indole-producing bacteria, while suppressing the expansion of pro-inflammatory and pro-oxidative microbial populations. Through restoration of microbial metabolic capacity, these interventions enhance endogenous antioxidant defenses and improve host redox balance. Currently, probiotics, prebiotics, postbiotics, fecal microbiota transplantation (FMT), and bacteriophage-based therapies represent the major microbiota-targeted approaches.

#### 5.1.1. Probiotics

Probiotics regulate intestinal microecological homeostasis through multiple mechanisms, including intestinal colonization, ecological niche competition, enhancement of epithelial barrier function, and production of bioactive metabolites. Through the production of metabolites such as SCFAs and tryptophan derivatives, probiotics can activate host antioxidant-related pathways, enhance ROS scavenging capacity, reduce low-grade inflammation, and contribute to intestinal redox equilibrium. Different strains within the same genus exhibit certain common antioxidant-related properties. In a rat stress model, combined intervention with commercial strains *Clostridium butyricum* CGMCC 0313-1 and *Bifidobacterium* infantis CGMCC 0313-2 upregulated intestinal Nrf2/HO-1 signaling, restored GSH and antioxidant enzyme activities, reduced MDA and protein carbonyl levels, and inhibited NF-κB expression and inflammatory factor release [154]. In another independent mouse enterotoxigenic *Escherichia coli* K88 infection model, *Clostridium butyricum* (swine-derived isolate) activated the p62-Keap1-Nrf2 signaling pathway, upregulated the expression of antioxidant genes such as Nrf2, HO-1, GSH-Px, and SOD, reduced serum MDA levels, and improved jejunal morphology and tight junction protein expression [155]. Different strains within the same genus also exhibit certain specificity in antioxidant-related capacity. One study first evaluated the in vitro antioxidant capacity of 27 *Lactobacillus plantarum* strains and found significant differences among strains. Subsequently, the researchers selected three strains with the highest (CCFM10), intermediate (CCFM242), and lowest (RS15-3) antioxidant capacity and validated them in vivo in a D-galactose-induced oxidative stress mouse model. The results showed that the antioxidant capacity of CCFM10 was also significantly superior to that of RS15-3 [156]. Another study compared the in vitro antioxidant capacity of *Lacticaseibacillus rhamnosus* LBUX2302 and *Lacticaseibacillus casei* from the same genus and found that the DPPH inhibition rate of L. casei was 15 times that of LBUX2302, but the hydroxyl radical scavenging rate of LBUX2302 cell-free supernatants (CFS) was significantly higher than that of L. casei (43%) [157]. These studies further demonstrate that probiotics help maintain host redox homeostasis, but the antioxidant-related performance of a single strain cannot represent its genus and requires differentiation.

Collectively, probiotics contribute to intestinal antioxidant defense through complementary mechanisms, including activation of Nrf2 signaling, enhancement of intracellular GSH availability, reinforcement of epithelial barrier integrity, and suppression of inflammatory responses [154]. Nevertheless, the efficacy of probiotics remains highly dependent on strain characteristics, host background, and ecological compatibility with the resident microbiota.

#### 5.1.2. Prebiotics

Prebiotics are selectively utilized substrates that promote beneficial microbial activity and confer health benefits to the host. Compared with direct microbial supplementation, prebiotics regulate the gut ecosystem by providing ecological advantages to specific functional microbial populations. Dietary fibers, including inulin and fructo-oligosaccharides, selectively promote the growth and metabolic activity of butyrate-producing bacteria, particularly members of the Lachnospiraceae and Ruminococcaceae families [158]. Clinical and experimental studies have demonstrated that supplementation with these substrates increases intestinal abundance of beneficial bacteria such as *Bifidobacterium* and enhances fecal butyrate concentrations, and improves microbial metabolic function, as reflected by increased butyrate production capacity, accompanied by improvements in OS-related parameters [159,160]. Polyphenols also exhibit microbiota-modulating properties. In addition to their intrinsic free radical-scavenging activity [161], dietary polyphenols such as ellagic acid and ellagitannins can be metabolized by intestinal microorganisms into bioactive metabolites, including urolithin A and urolithin B [162]. These microbial-derived metabolites have been reported to exert antioxidant-related effects through multiple mechanisms, including modulation of redox-sensitive signaling [163]. Meanwhile, polyphenol consumption is frequently associated with increased abundance of beneficial bacteria, including *Bifidobacterium* and *Lactobacillus* [164]. However, the effects of prebiotics are generally moderate and depend strongly on the baseline microbial composition of the host. Therefore, in diseases characterized by severe dysbiosis, more direct microbiota reconstruction strategies may be required.

#### 5.1.3. Postbiotics

According to the ISAPP consensus, postbiotics are defined as preparations of inanimate microorganisms and/or their components that confer a health benefit on the host [165]. Compared with live probiotics, postbiotics are less affected by gastric acidity, bile exposure, and competition with resident microbiota, offering improved stability and safety profiles, particularly for vulnerable populations [166]. Various postbiotic preparations have demonstrated antioxidant-related and barrier-protective effects. One study screened 6 strains from 88 *Lactobacillus* and *Bifidobacterium* strains to prepare postbiotics. In DSS-induced colitis mice, the postbiotic restored antioxidant indices such as SOD, CAT, GSH, and GPX, reduced MDA, downregulated pro-inflammatory factors, upregulated anti-inflammatory factors, and exerted antioxidant and anti-inflammatory effects through Nrf2 and NF-κB signaling pathways, with effects superior to the corresponding probiotics [167]. Heat-inactivated *Bifidobacterium longum* CECT-7347 whole-cell postbiotic enhanced oxidative stress tolerance in *Caenorhabditis elegans* and inhibited IL-8 secretion and NF-κB activation while maintaining tight junction protein ZO-1 expression in TNF-α-stimulated HT-29 intestinal epithelial cells, thereby alleviating inflammatory responses and intestinal barrier disruption [168]. The above studies demonstrate the antioxidant-related properties of postbiotics. However, because postbiotics lack the ability to colonize and continuously produce metabolites within the intestine, their beneficial effects generally require repeated administration. Future studies should further optimize their stability, delivery systems, and dose–response relationships.

#### 5.1.4. Fecal Microbiota Transplantation (FMT)

FMT involves transferring the complete microbial community from carefully screened healthy donors into the recipient intestine to reconstruct microbial ecosystem function. Compared with dietary interventions, FMT provides a more comprehensive approach for correcting severe microbiota disruption associated with oxidative stress-related diseases [169]. FMT has been reported to restore microbial metabolic capacity by enriching butyrate-producing bacteria, enhancing intestinal epithelial antioxidant responses, and reducing the abundance of facultative pathogenic bacteria such as Enterobacteriaceae [170]. Consistent with these findings, clinical evidence suggests that the efficacy of FMT in ulcerative colitis is linked to the recovery of butyrate-producing bacteria, which could drive clinical and endoscopic improvements in ulcerative colitis (UC) patients [171]. However, which components mediate the efficacy of FMT remains controversial. Fecal virome transplantation (FVT) and bacteriophage transfer studies suggest that non-bacterial components such as bacteriophages may contribute to the efficacy of FMT [172]. Similar discrepancies have been observed in broilers; some studies attribute the growth-promoting effects of FMT to the enrichment of lactic acid bacteria [173], whereas FVT studies confirm that non-bacterial components (virome and metabolites) can also improve feed conversion ratio and intestinal development [174], making the attribution of FMT effector components challenging. In addition, potential pathogenic bacteria and antibiotic resistance genes in donor feces may be transmitted to the recipient intestine via FMT, posing a threat to recipient safety [175], making rigorous donor screening essential. Overall, the mechanisms underlying FMT remain incompletely understood. Proposed mechanisms include restoration of microbial ecological networks, transfer of beneficial microbial metabolites, and the contribution of donor-derived bacteriophages or some gene transfer elements [176]. However, the specific components responsible for therapeutic efficacy require further investigation.

#### 5.1.5. Bacteriophages

Bacteriophages are bacteria-specific viruses that recognize their hosts through receptor-binding structures, including tail fibers, tail spikes, and baseplates. These structures interact with bacterial surface molecules such as LPS, outer membrane proteins, capsular polysaccharides, and pili, enabling highly selective targeting of specific bacterial species or strains without directly affecting host cells [177,178]. Compared with broad-spectrum antibiotics, which often disrupt microbial diversity and intestinal homeostasis [179], bacteriophage therapy provides a selective approach that eliminates pathogenic bacteria while preserving beneficial microbial communities [180]. By reducing the abundance of OS-promoting pathogens, bacteriophages may decrease the production of LPS and other inflammatory mediators, thereby indirectly alleviating intestinal oxidative injury. Furthermore, bacteriophages possess advantages including host specificity, self-amplification under permissive conditions, and the ability to disrupt bacterial biofilms [181,182]. Nevertheless, the emergence of phage resistance, host-range limitations, and interactions with resident microbiota remain important challenges requiring further optimization.

Collectively, microbiota-targeted remodeling strategies improve host redox homeostasis primarily by reconstructing microbial community structure and restoring beneficial metabolic outputs. However, their therapeutic effects depend on successful ecological integration of introduced microorganisms or substrates. As critical mediators of microbiota–host communication, EVs provide an alternative strategy by directly delivering antioxidant signals from microbes to host cells, representing an emerging direction for targeted regulation of intestinal OS.

### 5.2. Engineering Modification of EVs

EVs, including BEVs and host-derived EVs (e.g., Exos), possess several advantageous properties, such as intrinsic biocompatibility, nanoscale size, membrane protection of cargo molecules, and the ability to mediate intercellular communication across biological barriers. These characteristics make EVs potential delivery platforms for antioxidant molecules and regulatory factors [183]. Recent advances in synthetic biology, genetic engineering, and biomaterial-based modification strategies have enabled the development of engineered EVs with enhanced cargo-loading capacity and improved stability; however, their therapeutic efficacy for oxidative stress-associated diseases remains to be validated in clinical studies.

#### 5.2.1. Probiotic-Derived BEVs

Genetic engineering of probiotic strains provides an emerging strategy to generate BEVs enriched with specific antioxidant molecules. Compared with naturally produced BEVs, engineered probiotic-derived BEVs can achieve enhanced loading efficiency of functional proteins, peptides, or nucleic acids, thereby expanding their potential applications in redox regulation. A commonly used engineering approach involves fusing the coding sequence of a functional molecule, such as an antioxidant enzyme or regulatory protein, with genes encoding BEV-associated carrier proteins, including ClyA, OmpA, or Lpp-OmpA [183,184]. Following overexpression in engineered bacterial strains, the recombinant proteins can be preferentially incorporated into newly generated BEVs during vesicle biogenesis, resulting in functional cargo enrichment within the vesicles [185]. Compared with direct administration of recombinant proteins, engineered probiotic-derived BEVs may provide several advantages, including enhanced molecular stability, improved cellular uptake, and efficient intracellular delivery. However, challenges including optimization of cargo-loading efficiency, control of vesicle composition, and large-scale production remain to be addressed before clinical translation.

#### 5.2.2. Engineering of Host-Derived EVs

In addition to bacterial vesicles, host-derived EVs, particularly Exos, offer another potential strategy for antioxidant intervention. Exosome engineering typically involves large-scale culture of donor cells, including intestinal epithelial cells, mesenchymal stem cells (MSCs), or immune cells, followed by isolation, purification, and modification of secreted Exos with functional cargos [186]. MSC-derived Exos have attracted considerable attention because they naturally contain immunomodulatory and antioxidant-associated molecules, including miRNAs such as miR-146a, anti-inflammatory cytokine-related transcripts such as IL-10 mRNA, and antioxidant-associated proteins [187]. These intrinsic cargos enable MSC-derived Exos to suppress inflammatory responses, reduce oxidative injury, and inhibit ferroptosis in multiple disease models.

Further engineering strategies, including in vitro loading of antioxidant molecules, incorporation of functional nucleic acids, and surface modification with targeting ligands, can enhance cargo delivery efficiency and improve tissue-specific accumulation [188,189]. For example, Exos carrying Nrf2-related regulatory molecules have shown protective effects against oxidative injury in liver and kidney models after systemic administration [190]. Nevertheless, issues related to targeting specificity, immunogenicity, biodistribution, manufacturing consistency, and long-term safety require comprehensive evaluation before clinical application.

#### 5.2.3. Oral Delivery Strategies for EVs

Although EVs possess excellent biological compatibility, their oral application remains challenging due to degradation by gastric acid and bile salts, limited penetration through the intestinal mucus layer, and inefficient uptake by intestinal epithelial cells. To overcome these limitations, various biomaterial-based coating and delivery strategies have been developed, among which polymer-based microsphere encapsulation represents one of the most extensively investigated approaches [191]. Biomaterials such as chitosan and sodium alginate can be fabricated into microspheres or nanoparticles that encapsulate EVs or adsorb them onto their surfaces. These protective systems improve EV stability during gastrointestinal transit by reducing exposure to harsh digestive conditions. After reaching the intestine, particularly the relatively alkaline environment of the colon, the coating materials gradually degrade and release intact EVs, enabling local antioxidant and immunomodulatory effects [192]. Such delivery technologies may overcome the limitations of conventional EV administration and provide a foundation for the development of orally available EV-based therapeutics [193]. However, further studies are required to optimize encapsulation efficiency, control release kinetics, evaluate intestinal absorption mechanisms, and establish scalable manufacturing processes.

To facilitate comparison of the strength of evidence and translational potential across these intervention strategies, Table 2 summarizes their key mechanisms and outcomes, experimental models/study populations, evidence levels, major limitations, and representative references.

As shown in Table 2, diverse microbiota remodeling strategies exhibit respective pros and cons in restoring intestinal redox homeostasis. Sufficient and robust human-derived clinical evidence is still absent for most modalities.

## 6. Conclusions and Perspectives

The gut microbiota and host redox system form a dynamic and reciprocal regulatory network that is essential for maintaining intestinal homeostasis and systemic physiological balance. Increasing evidence indicates that gut microbial dysbiosis can disrupt redox equilibrium by reducing the production of beneficial antioxidant metabolites, promoting intestinal epithelial ferroptosis, impairing mucosal barrier integrity, and facilitating the dissemination of oxidative and inflammatory signals through gut–organ axes, thereby contributing to metabolic, neurological, and renal disorders. Conversely, host-derived signals, including epithelial EVs, may participate in reshaping microbial community structure and functional activity, further highlighting the bidirectional nature of microbiota–host redox communication. Recent advances in microbiota-targeted interventions, including probiotic and prebiotic modulation, postbiotic supplementation, FMT, bacteriophage-based approaches, and engineered EV delivery systems, have provided potential strategies for restoring redox homeostasis. These approaches not only improve microbial ecological balance but also modulate antioxidant-related signaling, reinforce intestinal barrier function, and attenuate systemic oxidative injury. Together, these findings provide a theoretical foundation for developing targeted microbiota-based therapies against OS-associated intestinal and multi-organ diseases.

Despite substantial progress, several fundamental challenges remain. First, causal relationships between microbiota alterations and host OS remain insufficiently defined. Most current studies are based on observational associations, making it difficult to determine whether microbial alterations drive oxidative injury or arise as a consequence of disease progression. Establishing causality requires more rigorous experimental approaches, including germ-free animal models, defined microbial consortia, genetic manipulation, and targeted metabolite intervention. Second, current analytical and intervention strategies are limited by insufficient spatiotemporal resolution. Conventional ex vivo assays cannot accurately capture dynamic changes in intestinal ROS generation, microbial metabolic activity, or EV-mediated communication within the native intestinal environment. In addition, in vitro models often fail to reproduce the complex interactions among microbiota, epithelial cells, immune components, and metabolites. For orally delivered microbial products or EV-based therapeutics, challenges including gastrointestinal degradation, epithelial transport barriers, biodistribution, and in vivo functional stability remain major obstacles. Furthermore, the lack of non-invasive human monitoring approaches and the limitations of currently available clinical samples, such as feces and intestinal biopsies, restrict direct evaluation of microbiota–redox interactions in patients.

Future studies should prioritize the establishment of standardized causal validation platforms, including synthetic microbial communities and multi-model experimental systems, to precisely define the functional contribution of individual microorganisms and metabolites. Advances in high-throughput screening technologies, spatial and temporal multi-omics integration, in vivo redox imaging, and real-time EV tracking will provide deeper mechanistic insights into microbiota–host communication. Meanwhile, the development of engineered probiotics, targeted EV delivery systems, and gut–organ axis-based combination interventions may facilitate the transition from mechanistic discovery to targeted microbiota therapeutics. Overall, a comprehensive understanding of gut microbiota–host redox interactions will not only reveal fundamental principles governing intestinal and systemic homeostasis but also provide innovative strategies for the prevention and treatment of OS-related diseases.

## Figures and Tables

**Table 1:** Evidence sources and key limitations for gut microbiota–host redox crosstalk.

Section	Mechanism	Evidence Source	Key Limitations	Representative Refs.
Section 3.1	EV/BEV-mediated communication	In vitro (Caco-2 cells); animal (HFD mice, DSS colitis); milk-derived Exos	Endogenous epithelial Exos insufficiently studied	[103,106,107,111,114,115]
Section 3.2	Microbiota-regulated intestinal epithelial ferroptosis	Animal (germ-free mice, DSS colitis); in vitro (cell models)	GPX4-independent pathways poorly linked to microbial metabolites	[117,118,121,123]
Section 3.3	Microbial metabolic reprogramming	Animal (DSS colitis); in vitro; human observational (aging cohorts)	Microbial composition does not necessarily reflect function; metabolic flux data are limited	[20,128,129,130,131,132]
Section 4.2.1	Gut–liver axis	Animal (LPS liver injury mice); human observational (MASLD patients)	Causality difficult to establish	[143,144,146]
Section 4.2.2	Gut–brain axis	Animal (heart failure mice); in vitro (microglial models)	Human neurodegenerative causality unproven	[147,148,149]
Section 4.2.3	Gut–kidney axis	Animal (CKD models); human observational (CKD patients)	Directionality uncertain	[150,151,152]

Abbreviations: EV, extracellular vesicle; BEV, bacterial extracellular vesicle; HFD, high-fat diet; DSS, dextran sulfate sodium; GPX4, glutathione peroxidase 4; MASLD, metabolic dysfunction-associated steatotic liver disease; CKD, chronic kidney disease.

**Table 2:** Comparison of gut microbiota-targeted intervention strategies for redox regulation.

Intervention Type	Key Outcomes/Effects	Evidence Source	Main Limitations	Representative References
Probiotics	Enhance antioxidant-related defense, inhibit inflammation, and strengthen tight junctions	In vitro (cell models); animal (rat stress model; ETEC K88 mice)	Strong strain specificity; host background and ecological compatibility	[89,154,155]
Prebiotics	Promote butyrate production, activate antioxidant-related pathways, increase beneficial bacteria, and improve microbial metabolic function	In vitro (cultures); human (small-scale human studies, some RCT evidence)	Baseline microbiota-dependent; polyphenols are not classically prebiotics	[158,160,161,162,163,164]
Postbiotics	Restore antioxidant-related indices, reduce lipid peroxidation, inhibit inflammation, and maintain barrier function	In vitro (HT-29 cells, Caenorhabditis elegans); animal (DSS colitis mice)	No colonization; repeated dosing needed; stability, delivery, dose, and standardization unclear	[166,167,168]
FMT	Restore microbial ecosystem, enrich butyrate-producing bacteria, and reduce pro-inflammatory bacteria	Human (UC patients); animal (broilers); in vitro (FVT/phage transfer studies)	Donor pathogen/ARG risk; unclear effector components; mechanisms incompletely understood	[176]
Bacteriophages	Selectively eliminate pathogens, reduce inflammatory mediators, and disrupt biofilms	In vitro (studies)	Phage resistance; narrow host range; unclear interactions with resident microbiota	[177,178,179,180,181,182]
Engineered EVs	Deliver antioxidant-associated and anti-inflammatory cargoes, inhibit ferroptosis, and enable colon-targeted release	In vitro (cells); animal (liver/kidney models, intestinal inflammation models); oral delivery systems	Scale-up, cargo loading, safety, and oral delivery remain unresolved	[183,184,186,187,188,189,190,191,192,193]

Abbreviations: ETEC, enterotoxigenic *Escherichia coli*; DSS, dextran sulfate sodium; UC, ulcerative colitis; ARG, antibiotic resistance gene; FMT, fecal microbiota transplantation; FVT, fecal virome transplantation; RCT, randomized controlled trial.

## Data Availability

No new data were created or analyzed in this study. Data sharing is not applicable to this article.
